# A Possible Relationship between Peri-Implantitis, Titanium Hypersensitivity, and External Tooth Resorption: Metal-Free Alternative to Titanium Implants

**DOI:** 10.1155/2021/8879988

**Published:** 2021-01-22

**Authors:** Andrea Enrico Borgonovo, Rachele Censi, Virna Vavassori, Mauro Savio, Dino Re

**Affiliations:** ^1^Unit of Esthetic Dentistry, Istituto Stomatologico Italiano, University of Milan, Milan, Italy; ^2^Unit of Periodontology, Istituto Stomatologico Italiano, University of Milan, Milan, Italy; ^3^Head, Unit of Esthetic Dentistry, Istituto Stomatologico Italiano, Department of Biomedical, Surgical and Dental Sciences, University of Milan, Milan, Italy

## Abstract

Titanium dental implant surface does not remain unaltered but may corrode and release ions or particles which trigger soft and hard tissue damage. Titanium may induce clinically relevant hypersensitivity in patients chronically exposed. A 56-year-old female patient presented peri-implantitis around a single titanium implant positioned three years earlier. Despite nonsurgical therapy, a rapid bone loss associated with pain and swelling occurred, and adjacent teeth presented external resorption. Compromised teeth were removed, and three titanium implants were inserted. Six months later, the patient complained about high mucosa sensitivity and implant exposure. At clinical and radiographic examinations, tissue inflammation and vertical bone loss involved the new implants and the process of external resorption affected the teeth. The blood test confirmed titanium hypersensitivity. Titanium implants were removed, and 5 zirconia implants were placed. No sign of bone loss or tooth resorption was recorded at clinical and radiographic control during 18 months of follow-up.

## 1. Introduction

Extraction of permanent teeth is carried out for several reasons, including caries, periodontal disease, fractures, orthodontic/prosthetic purpose, and extensive internal or external tooth resorption. Pathologic resorption of teeth has a multifactorial etiology although many aspects remain unclear and can lead to tooth loss [1].

The use of dental implants has become a predictable strategy for replacing missing teeth, and the satisfactory results reported by numerous clinical studies have determined an enormous development of implantology [2]. Currently, the success rate of implants is around 95% in the maxilla and 97% in the mandible after 10 years of follow-up period [3, 4].

However, the increase in the demand for implants is associated with a growing need for longer term results, and, despite the high success rates, implant failures may occur due to biological or biomechanics complication [5].

Among the biological complications, peri-implantitis plays an important role. The American Academy of Periodontology defines peri-implantitis as an inflammatory process around an osseointegrated implant, including both soft tissue inflammation and progressive loss of supporting bone beyond biological bone remodeling [6]. Clinically, peri-implantitis sites exhibit signs of inflammation and, in particular, increased probing depths and/or recession of the mucosal margin, bleeding on probing and/or suppuration, and radiographic bone loss [7, 8].

The prevalence of peri-implantitis has grown enormously over the last decades: recent data report a percentage of implant exhibiting peri-implantitis around 15%-20% after 10 years [9]. Considering the clinical relevance of this disease, research activity has been addressed to identify the etiopathogenesis of peri-implantitis in order to recognize effective protocols for prevention and treatment. Nowadays, there is a lack of consensus on the exact etiology of peri-implantitis and subsequent pathological process [10, 11], although many studies describe different potential mechanisms for peri-implant bone loss, including the microbial biofilm and metal ion/particle release [12]. Titanium degradation products in the form of microparticles or ions may infiltrate the peri-implant tissues and peri-implant bacterial plaque and trigger an inflammatory response with bone resorption, suggesting implications on the pathogenesis of peri-implantitis [13]. Moreover, the literature shows that allergic reactions and hypersensitivity to metal are not uncommon findings; in fact, delayed-onset T-cell-mediated metal hypersensitivity is reported in 12% to 17% of the general population [14].

The emerging limits of titanium have prompted the research to focus on alternative materials. Among the new generation of ceramics in the dental field, zirconia presents excellent aesthetic characteristics [15], a low tendency of plaque adhesion on implant surface [16], excellent biocompatibility [17], good osseointegration, and biomechanics [18]. In addition, zirconia implants have characteristics similar to those of titanium and are frequently used in implant-prosthetic rehabilitations.

The aim of this case report is to describe and analyze the failure of two implant-prosthetic rehabilitation with titanium implants due to a synergy between peri-implantitis and titanium hypersensitivity and the success of a metal-free implant prosthetic rehabilitation in the same patient. In association with implant failure, pathologic external resorption in adjacent teeth was identified.

## 2. Case Presentation

**Figure 1:**
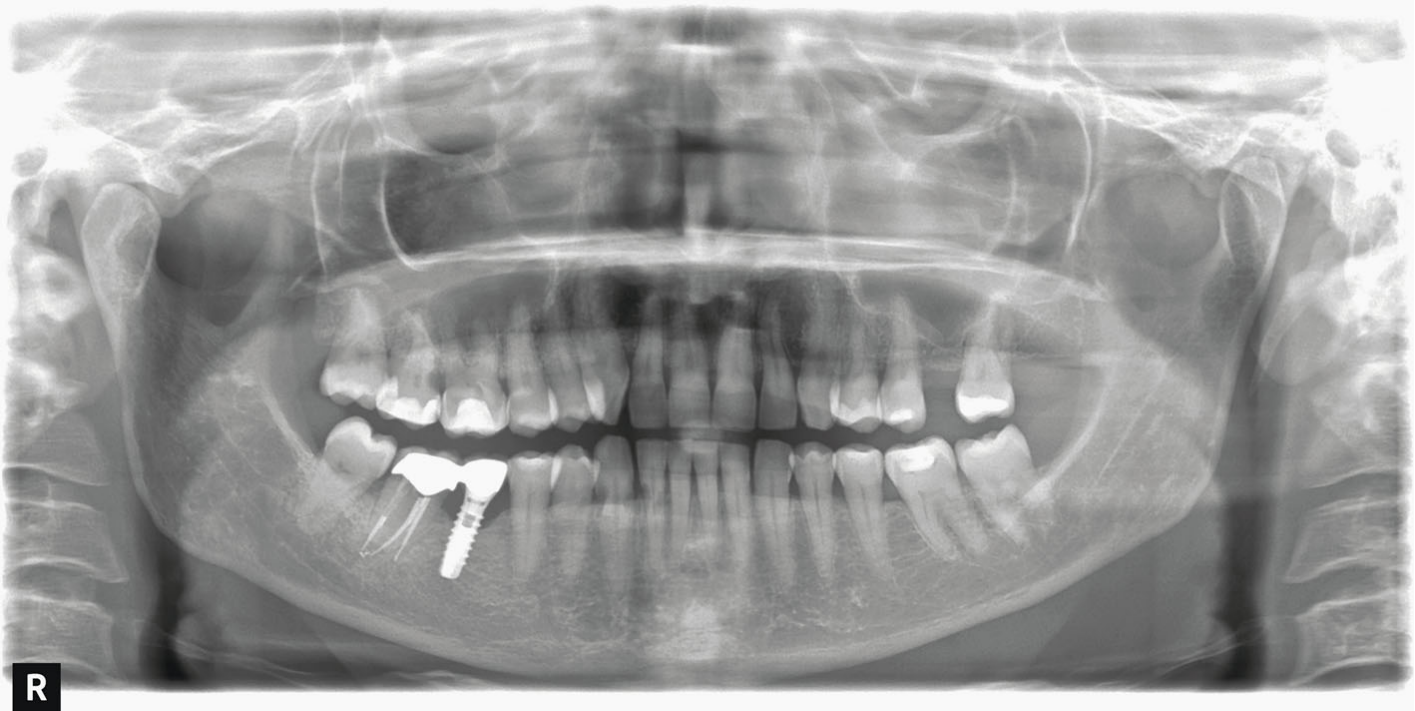
Implant 4.6 with initial signs of peri-implantitis.

A 56-year-old female patient presented at the Department of Esthetic Dentistry, Istituto Stomatologico Italiano, University of Milan, Italy, after being treated in a private clinic. She came to our attention with a partial edentulism in the mandible, consequent to multiple implant failures. Thanks to her medical record, it was possible to reconstruct her history. The patient went to the private clinic for pain and swelling around the implant positioned in zone 4.6 three years earlier. She had a medical history of hypertension, treated with a *β*-blocker therapy and allergies to pollen and dust. The patient denied any history of tobacco smoking and alcoholism. The last dental check-up visit was done about two years ago. Even if the patient had many restorative and prosthetic treatments, no previous history of periodontitis was detected. At clinical evaluation, peri-implant mucosa appeared swelling and redness and the probing revealed bleeding and a probe depth of 6 mm, buccally and 5 mm, lingually. The radiographic image (OPT) showed a bony defect with a crater-like shape around implant 4.6 and cervical decay on teeth 2.6 and 2.7 (Figure 1). A diagnosis of peri-implantitis was supposed, and the patient was treated with nonsurgical laser therapy. Moreover, she was recommended to maintain excellent oral hygiene standards, attend regular professional maintenance, and treat the decay.

**Figure 2:**
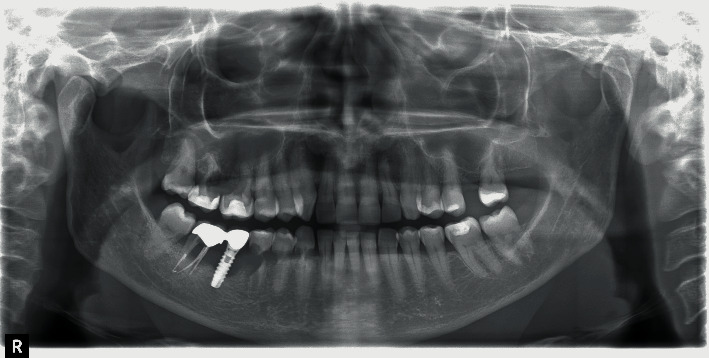
Tooth resorption and worsening of peri-implantitis.

**Figure 3:**
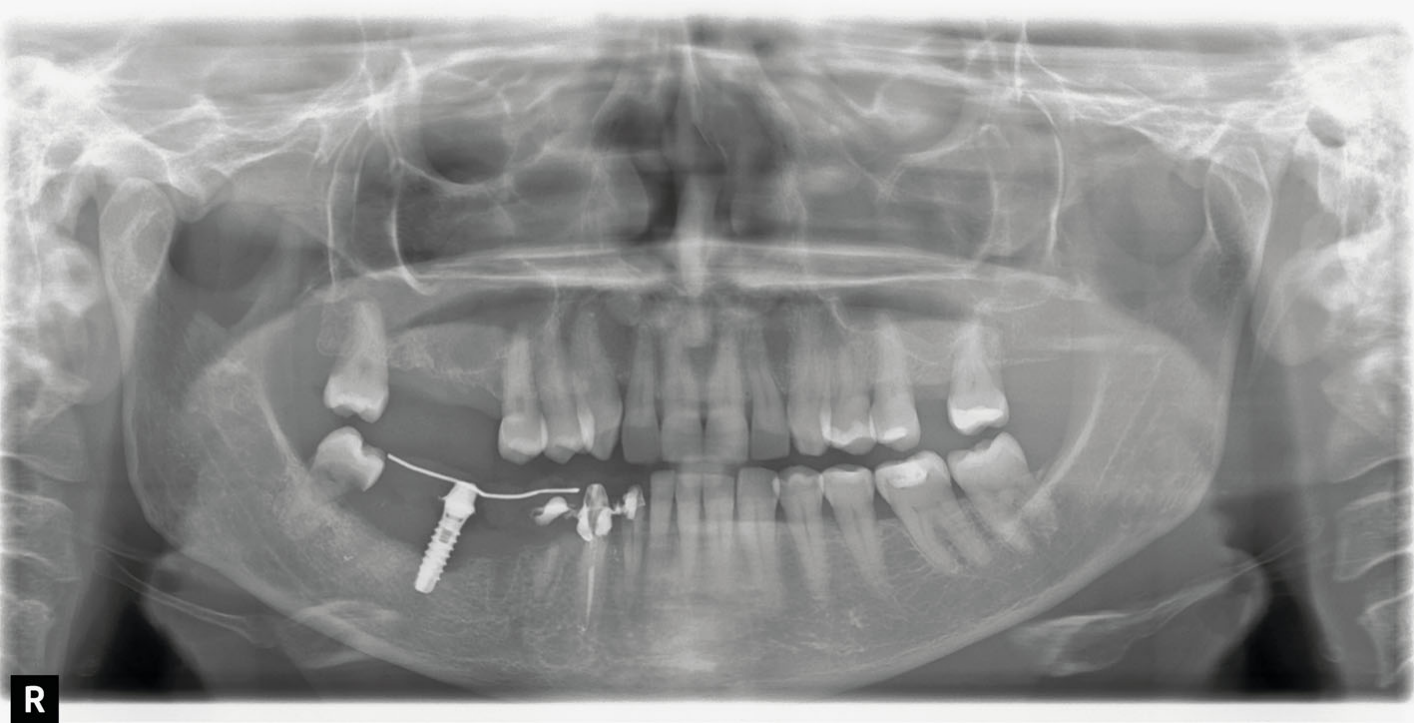
After extraction of compromised teeth, a provisional prosthesis was positioned.

**Figure 4:**
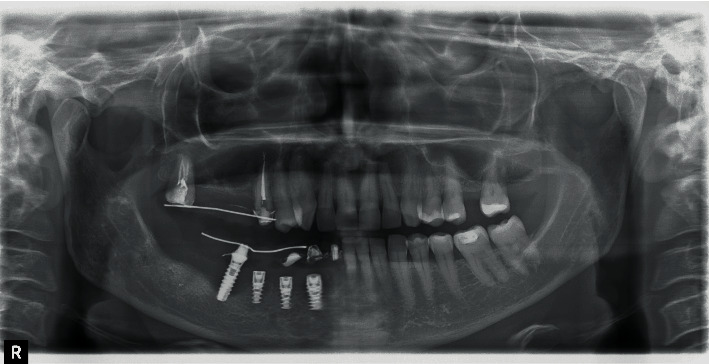
Insertion of three titanium implants.

Some months later, the problem has not been solved; the patient returned with pain and swelling around implant 4.6. The orthopantomography showed an increased bone loss which resulted in a deep vertical bony defect. Moreover, at an X-ray picture, a severe resorption of the teeth close to the implant in both of the jaws was observed (Figure 2). The compromised teeth were extracted but the implant was temporarily maintained to support a provisional fixed prothesis (Figure 3). Four months after dental extractions, three new titanium fixtures were inserted and loaded with a fixed prothesis. The patient denied implant 4.6 removal because it was stable and no longer symptomatic (Figure 4).

**Figure 5:**
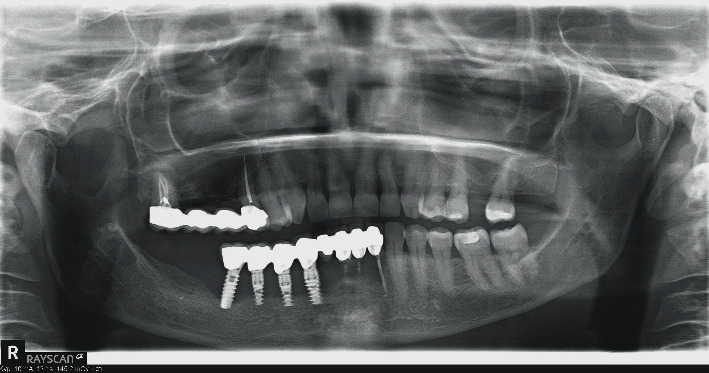
Six months after implant surgery: peri-implantitis affects new implants and tooth resorption involved the teeth up to the 3.3.

Six months later, the patient complained about high mucosa sensitivity and implant exposure. At clinical and radiographic examinations, tissue inflammation and vertical bone loss involved the new implants and the process of external resorption affected the teeth up to the 3.3 (Figure 5).

**Figure 6:**
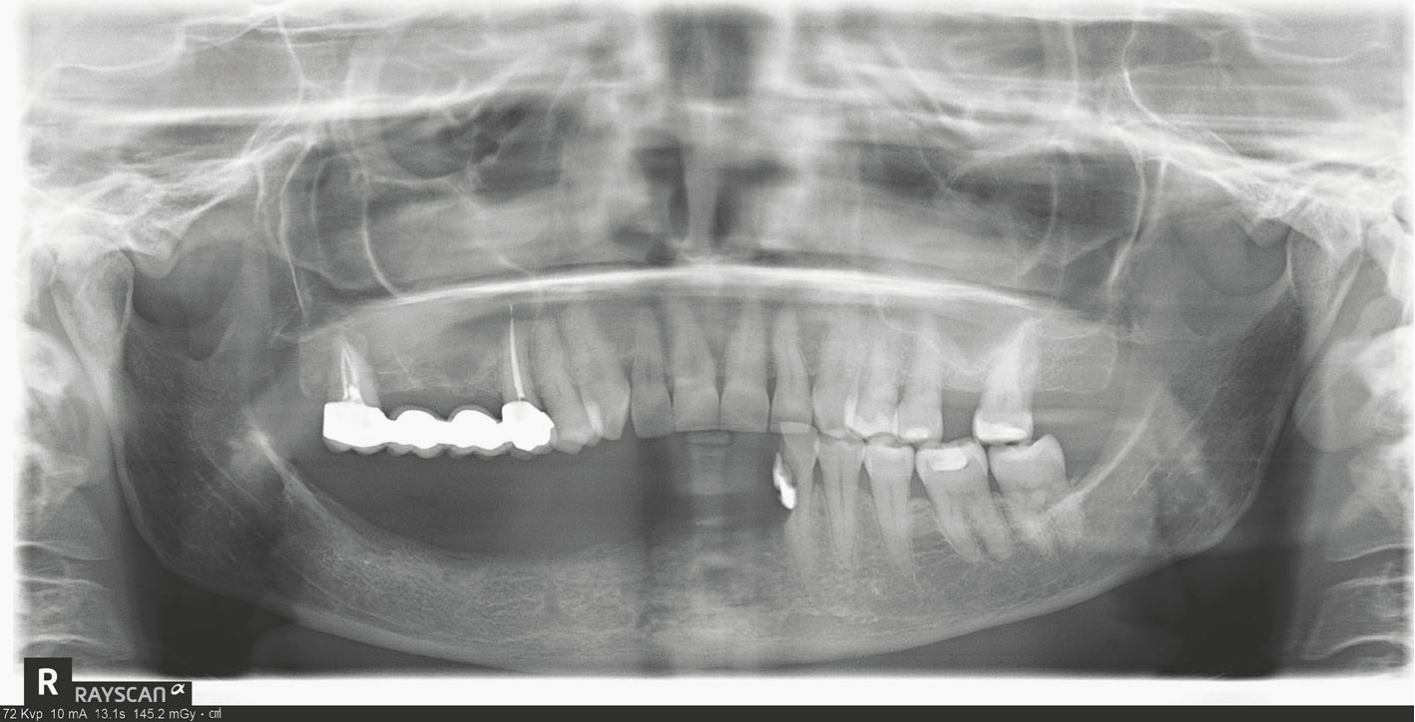
MELISA confirmed titanium hypersensitivity: implants were removed.

**Figure 7:**
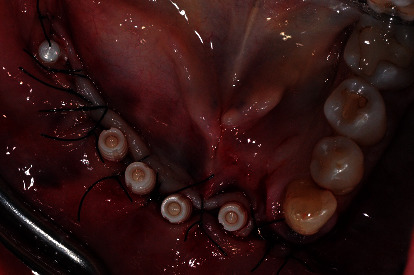
Surgical phase: five one-piece zirconia implants were placed.

**Figure 8:**
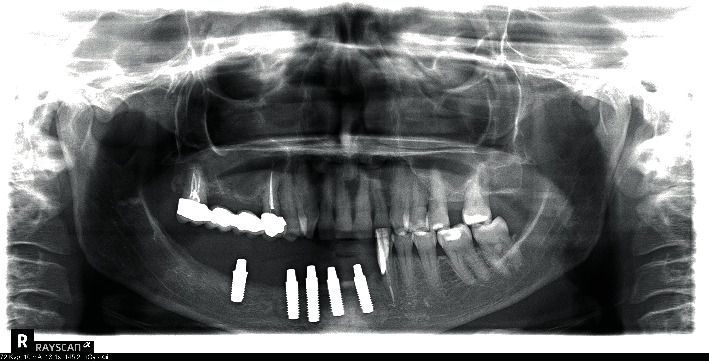
OPT after implant insertion.

**Figure 9:**
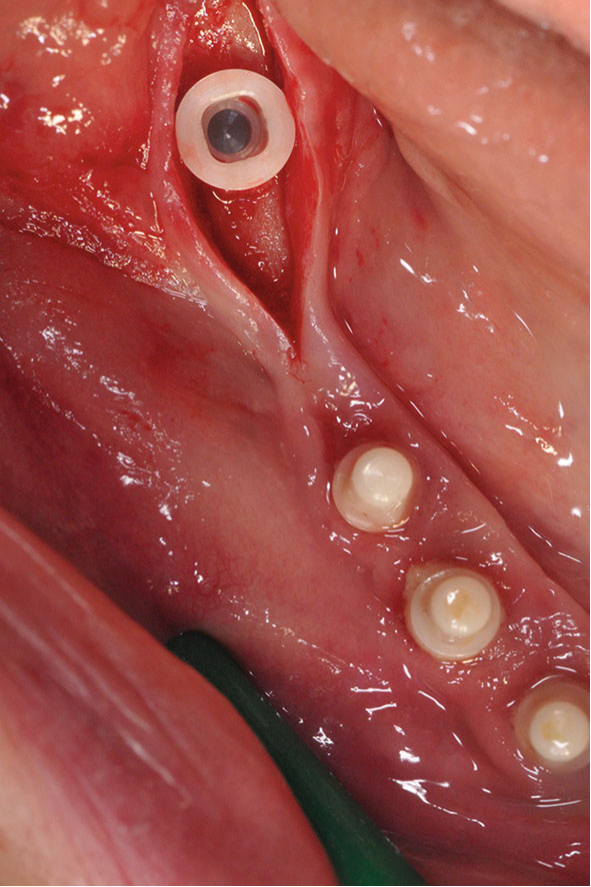
Placement of a two-piece zirconia in the posterior area after implant failure.

**Figure 10:**
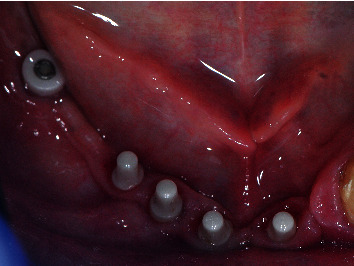
Clinical view before prosthesis positioning.

**Figure 11:**
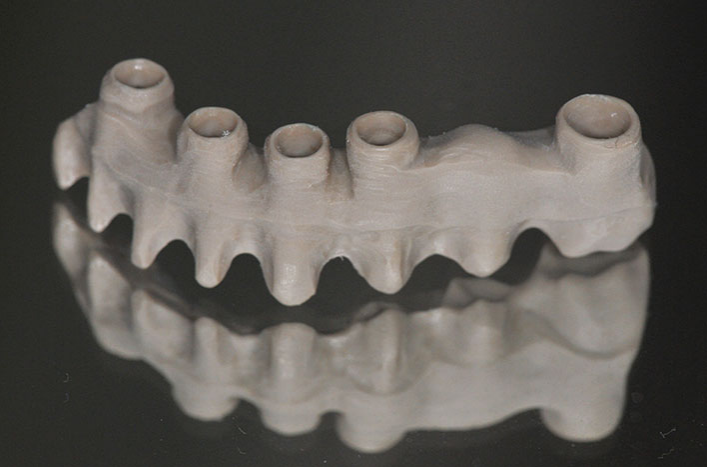
Modified PEEK framework.

**Figure 12:**
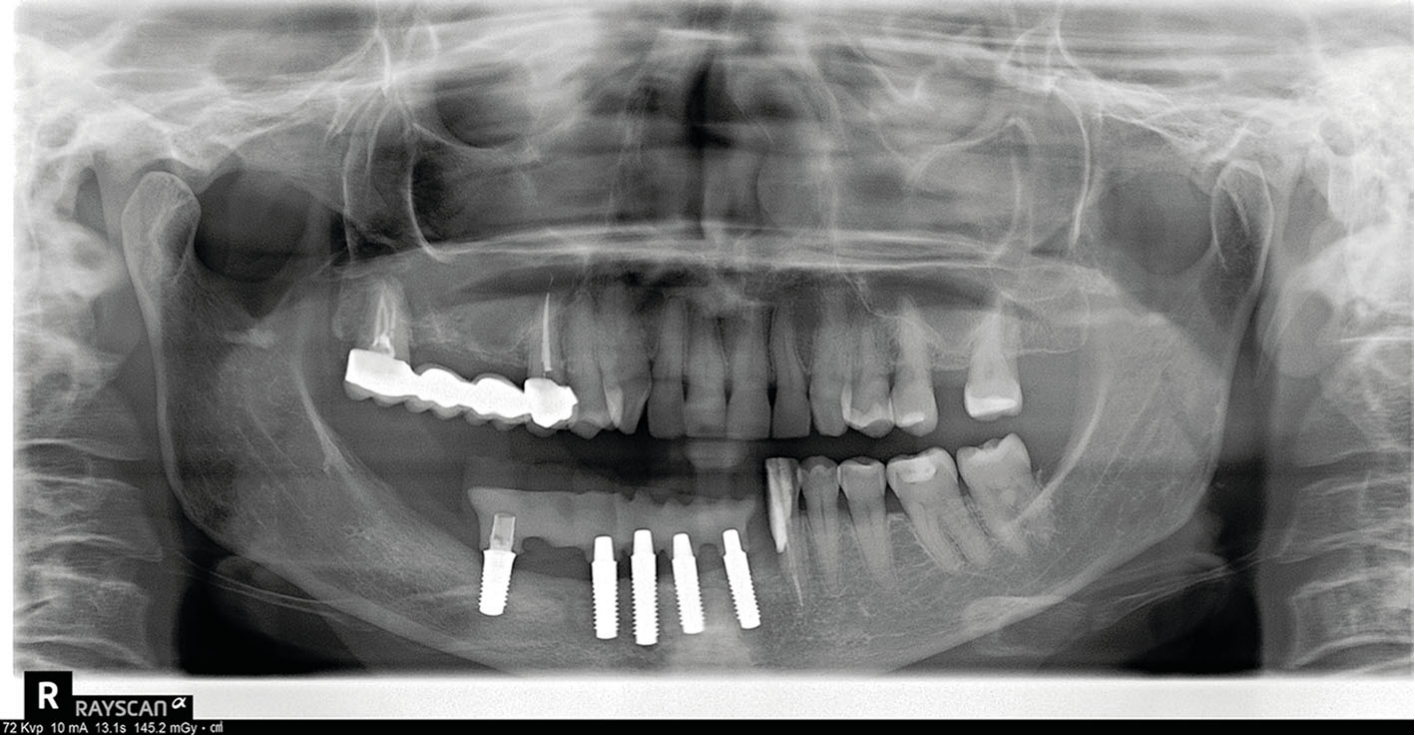
Rx control at 18 months of follow-up.

A biopsy was performed taking a sample of cortical and medullary bone to check for bone disorder. The result did not show any kind of bone lesion or disease and even the bacterial culture was negative. The patient was referred from the private clinic to the Department of Esthetic Dentistry for clinical assessment. Medical tests were programmed in order to investigate her condition. Standard blood tests revealed an increased number of eosinophils and so titanium intolerance was supposed. The MELISA (Memory Lymphocyte Immunostimulation Assay) test was performed and confirmed titanium hypersensitivity. It was decided to remove all the titanium fixtures and the problematic teeth. Despite the removal of the titanium implant, the patient referred to high mucosa sensitivity for a long period (Figure 6). Subsequently, after nine months, when algic symptoms disappeared, five one-piece zirconia implants were inserted, four in the anterior jaw and 1 in the right molar region (Straumann PURE Ceramic Monotype, Straumann) (Figures 7 and 8). Although a submerged healing was advisable, one-piece zirconia implants were chosen because in the anterior area the bone volume was reduced, and two-piece zirconia implants are available in diameters from 4.1 mm. On the other hand, the one-piece ceramic system offers narrow-diameter zirconia implants (3.3 mm). The posterior implant was lost two months after the loading and then substituted with a two-piece zirconia fixture (Straumann PURE Ceramic, Straumann) (Figure 9). The fixtures were loaded with a fixed metal-free prosthesis made of modified PEEK (Biohpp, Bredent medical, Senden Germany) (Figures 10 and 11). During the follow-up period, the patient did not refer to any symptoms of peri-implantitis or other problems, and after 18 months from surgery, the clinical-radiographic exams showed the success of the metal-free implant prosthetic rehabilitation. In particular, peri-implant tissues appeared healthy, and the X-ray pictures confirmed the absence of marginal bone loss around implants and no sign of tooth resorption (Figure 12).

## 3. Discussion

Modern implantology is based on the concept of osseointegration proposed by Branemark in 1977 [19]. Osseointegration refers to a direct structural and functional connection between living bone and the surface of a “load-bearing” (titanium) implant [20]. This phenomenon begins with implant placement into alveolar bone and takes up to several weeks. During this period, a cascade of healing events occurs resulting in direct bone contact with the implant surface. If initially, the interest was mainly addressed to the interactions that the bone had around the implant; recently, the attention was directed to the possible influence of metal ions or particle release from implant surface into surrounding tissues [21]. Moreover, in the last decades, an increase in the number of peri-implantitis has required investigations about its causes and risk factors.

In literature, many studies have detected the release of titanium ions and particles from the implant surface with the consequent activation of an inflammatory response [22, 23]. Dissolved particles can accumulate in the peri-implant tissues or can be released into the blood and lymphatic circulation and found in regional lymph nodes and internal organs [24]. Several hypotheses have been proposed about the release mechanisms. According to mechanical theory, titanium particles can be released from implant-abutment connection or during implant insertion or decontamination [25]. The chemical mechanism involves saliva, bacteria, and chemicals that potentially dissolve the titanium oxide layer of implants and trigger a process defined as biocorrosion [26]. Recent studies report high levels of dissolved titanium in submucosal plaque of implants with peri-implantitis compared with healthy implants, suggesting a relation between titanium release and peri-implantitis [27]. Reports suggest that peri-implantitis bacteria trigger an inflammatory response and cause electrochemical alteration of the titanium surfaces that include disruption of the titanium oxide layer and release of titanium particles and ions, influencing the pathogenesis of peri-implant bone loss. Moreover, corrosion may be enhanced by some everyday chemical agents, such as acid fluoride solutions [28].

The metal particles and ions released from implant surfaces act as foreign bodies and stimulate inflammatory reactions through the activation of a number of mediators. In particular, phagocytic cells (macrophages and multinucleate giant cells) phagocytize metal particles and promote an immune response through the release of proinflammatory cytokines and grown factors that induce inflammatory reaction and osteoclastogenesis with consequent bone resorption [29]. Titanium ions influence the expression of RANKL (Receptive Activator of Nuclear factor Kappa-B Ligand) and osteoprotegerin (OPG) in osteoblastic cells, promoting osteoclast activity.

This immune response is also strictly connected with titanium hypersensitivity even if the exact cellular pathway involved has not yet been clarified. Probably, metal ions bind to native proteins and form haptenic antigens that trigger the degranulation of mastocytes and basophiles and develop type IV hypersensitive reactions [30].

Hypersensitivity to metal in the general population is frequent, affecting up to 15% of patients, and nickel is the biggest offender, followed by cobalt and chromium [31]. However, recent data suggest an increased incidence of hypersensitivity to titanium for patients with dental implant and orthopedic arthroplasties. Diagnostic technique for type IV sensitivity reaction to potential allergens is commonly based on patch testing, but in the case of titanium, hypersensitivity is not predictable [32].

Titanium hypersensitivity is detected with MELISA that measures lymphocytic proliferation after antigen-specific activation. From a general point of view, the confirm of type IV hypersensitivity requires the avoidance of responsible agent and, for this reason, in the field of implantology, patients with clinical symptoms of hypersensitivity and elevated MELISA levels may undergo surgical removal of titanium implants and alternative material, such as ceramics, must be considered for rehabilitation of edentulous areas.

Oliva et al. describe a case report of a full-mouth oral rehabilitation with zirconia implants in a titanium allergy patient. No complications were recorded after 3 years of follow-up [33].

The presented case may be an example of titanium hypersensitivity probably potentiated by peri-implantitis, even if the report presents some limits that might act as possible cofactors in implant failure. It is well-known that there are several problems connecting a tooth with an implant, including tooth intrusion with consequent loss of crown retention. Moreover, exposed implant surface and continued contamination can lead to peri-implantitis [34].

The medical history of the patient suggested allergies to dust and pollen but not to metals. However, it is known that an allergy can develop at any time in life. Probably, the first single titanium implant rehabilitation and the development of peri-implantitis that commonly affect implants have triggered immune response and consequent hypersensitivity. After implant failure, three titanium implants were placed developing a more intensive allergic reaction. The peculiarity of this case resided not only in the implant failure consequent to titanium hypersensitivity but also in the phenomenon of external resorption of the adjacent teeth. Pathologic tooth resorption is associated with an abnormal stimulation of the RANKL system which is present not only in osteoblasts but also in odontoblasts, cementoblasts, and ameloblasts [35]. As reported, the release of titanium particles and ions influence RANKL expression, suggesting a connection between titanium hypersensitivity and external tooth resorption.

However, after the second implant failure, it was decided to proceed with a metal-free rehabilitation positioning 5 zirconia implants. Zirconia implants appeared well integrated without marginal bone loss, and no sign of pathological tooth resorption was detected at 18 months of follow-up.

Zirconia has some advantages over titanium implants: although the success and survival rates of zirconia and titanium implants are similar, some studies have reported that zirconia has more biocompatibility as compared to titanium, as the latter produces corrosion products at the implant site [36]. However, further investigations about titanium release and its connections with peri-implantitis, hypersensitivity, and tooth resorption are recommended.

Patients detected with hypersensitivity by MELISA should be treated with zirconia implants and metal-free prosthesis. For this reason, zirconia implants may represent a valid alternative to titanium even if further studies are still needed [37].
